# Long-term safety and efficacy of non-absorbable abdominal mesh for apical prolapse surgery: A 10-year experience at Tertiary Care Hospital

**DOI:** 10.12669/pjms.39.2.6689

**Published:** 2023

**Authors:** Samia Aijaz, Novera Chughtai, Urooj Kashif, Summera Malik

**Affiliations:** 1 Samia Aijaz, Clinical Fellow Urogynecology and Pelvic Reconstructive Surgery, Department of Obstetrics & Gynecology, Aga Khan University Hospital, Karachi, Pakistan; 2 Novera Chughtai, Clinical Fellowship Urogynecology AKUH, Assistant Professor, Department of Obstetrics & Gynecology, Aga Khan University Hospital, Karachi, Pakistan; 3 Urooj Kashif, Assistant Professor, Department of Obstetrics & Gynecology, Clinical Fellowship Urogynecology AKUH, Aga Khan University Hospital, Karachi, Pakistan; 4 Summera Malik, Clinical Fellow Urogynecology and Pelvic Reconstructive Surgery, Department of Obstetrics & Gynecology, Aga Khan University Hospital, Karachi, Pakistan

**Keywords:** Apical Prolapse, Hysteropexy, MESH, SacroHysteropexy, SacroColpopexy

## Abstract

**Objectives:**

To evaluate the complications of apical prolapse correction with abdominally placed mesh and to assess the long-term efficacy of Sacrohysteropexy and Sacrocolpopexy.

**Method:**

A retrospective cohort study was conducted at the Department of Gynecology and Obstetrics, Aga Khan University Hospital. All women who underwent apical prolapse surgery using abdominal mesh from January 2010 to December 2019 at AKUH were included. Patients with missing routine follow up visits and incomplete data up to one year post op were excluded. Patient notes were reviewed, and subjective and objective success and complications were analyzed. Safety was measured by incidence of intra, early and late postoperative complications and mesh-related complications of both procedures at two weeks, six months, twelve months, postoperatively.

**Results:**

A total of 69 cases were retrieved from the database with a mean age of 46.97 ± 13.86 years. It was found that 14 (20.3%) patients had wound infection while six (8.7%) patients developed urinary tract infections. In a median follow-up of 12 months, three patients developed mesh erosion as a complication, with an incidence of 4.3%. Two required surgical excision of the mesh and the third was successfully managed conservatively with topical estrogen and oral antibiotics. Extremely significant improvements were observed in POPDI-six scores six months postoperatively (p=0.0001).

**Conclusion:**

The present study signifies the use of abdominally placed mesh in patients with pelvic organ prolapse indicating significant improvement in Pelvic Organ Prolapse-associated symptoms postoperatively.

## INTRODUCTION

Herniation of the uterus or vaginal vault (after hysterectomy) through the vagina is known as uterine or vaginal vault prolapse (VVP). This also known as apical prolapse.^1^ When conservative measures fail, all surgical options should be considered and discussed with patients.

Sacrohysteropexy (SHP) is a uterus-sparing surgical procedure that has gained more favor and has demonstrated satisfactory medium-term outcomes. Compared to hysterectomy, hysteropexy allows for higher apical suspension and greater vaginal length. Hysteropexy has not only shown better outcomes in the medium-term but also less intraoperative adverse events such as bleeding and pain.^2^ Mesh use for correction of apical prolapse has been extensively studied, but there is still need for long-term, well-designed cohort studies in view of recent controversy of mesh related complications.^3^

The exact prevalence of VVP is undetermined but cited as 0.2% to 43%.^4^ Many corrective procedures for VVP are at the surgeon’s disposal but Sacrocolpopexy (SCP) is the gold standard. SCP is correlated with positive outcomes, in contrast to other procedures, such as reduced recurrence rate, rate of recurrent procedures, and risk of stress urinary incontinence following surgery.^5^ Mesh repair of pelvic organ prolapse (POP) demonstrates a reduced recurrence rate, in contrast to traditional repair which has a higher rate of anatomic recurrence i.e. 30%.^6^ Present media attention to the negative experiences of women after mesh reconstructive surgery has led to reservations regarding abdominal mesh placement. It has become questionable whether mesh procedures are entirely safe and effective.

In abdominal mesh placement for AP, a mesh may come in direct contact with either abdominal viscera or the vagina. This may lead to erosive injury. In abdominal Sacrocolpopexy (ASC) the rate of mesh-related erosion is cited as 2% to 11%. ASC is also associated with a 2% risk of serious adverse events.^7^ After SCP contact between mesh and the vagina was 0.7% as per one study^8^ and 2% as per another.^9^ In contrast, SHP did not result in cases of mesh exposure in the long-term. Complication rate was 1.8%.^10^

In the UK since early June 2018, all Trans abdominal mesh interventions are only authorized subject to “high vigilance” with stringent regulation. Tapes for POP and transvaginal mesh for SUI are considered suspended (on “pause”) and cannot be utilized.^10^ In view of the recent debate regarding use of mesh for POP, we conducted a retrospective analysis of surgeries performed at AKUH for POP using polypropylene mesh for both vault and uterine prolapse, In our population, there is very little data on such mesh-related prolapse surgeries and mesh-related complications, and there is a lack of knowledge on long-term outcomes.^11^ The present study evaluated the complications of apical prolapse corrected with abdominally placed mesh and to assess the long-term safety and efficacy of Sacro hysteropexy and Sacro colpopexy.

## METHODS

A retrospective cohort study was conducted at the department of Gynecology and Obstetrics, Aga Khan University Hospital. A non-probability convenient sampling technique was employed. All women who underwent apical prolapse surgery using abdominal mesh which is SHP for uterine prolapse and SCP for vault prolapse from January 2010 to December 2019 at AKUH were included. Patients with missing routine follow up visits and incomplete data up-to one year post op were excluded.

After exemption from Ethical review committee approval #2022-7145-20771 from AKUH, data was collected by using the international classification of disease, 10^th^ revision, clinical modification (ICD-10). Procedure codes 69.22 and 70.77 were used to identify the women who underwent SCP and SHP for apical prolapse for 10 years duration. Hence file notes were reviewed, and subjective and objective success, objective assessment of POP was done using POP-Q scale at preoperative for baseline and every postoperative visit (6 weeks, 6month and 12^th^ month) and subjective assessment by using POPDI-6 at every postoperative follow-up visit, recorded complications were analyzed. Safety was measured by incidence of intra operative complications which included estimated blood loss, bowel injury, operative time, need of blood transfusion and severity of adhesions, for all our cases, the diagnosis of pelvic adhesions was only confirmed during the surgery and not from preoperative assumptions, Adhesions were divided into four classes according to the Operative Laparoscopy Study Group (OLSG) classification and adhesion scoring system proposed by Luciano et al. as follows 0,no adhesion, one filmy adhesion, blunt dissection, two strong adhesion, sharp dissection, 3 very strong vascularized adhesion, sharp dissection, damage hardly preventable^12^,^13^, early complications encountered within two weeks and late postoperative complications including mesh related complications including exposure, erosion, infection, rejection, migration of both procedures at one to six months and six to twelve months, postoperatively. At our setup Every woman postoperatively assessed on urogynecology assessment form including specific questions about quality of sexual life in the past four weeks, including thorough nonjudgmental sexual history with open ended questions

The success of surgery was assessed by documented subjective improvement in the initial symptoms of POP. Postoperative subjective success was assessed by using and comparing the documented pelvic organ prolapse distress inventory (POPDI-six) questionnaire at pre-operative and follow up visits at 1-6 months and 6-12 months postoperatively. The objective evidence of the degree of prolapse was determined using POP-Q staging. Postoperatively patients were regarded as cured if they had POP one cm above hymen for stage 3 and above and stage zero for patients with initial lesser stage of POP.

Patient’s identifiers like name, medical record numbers and addresses were not documented to ensure confidentiality and anonymity. Data was entered and analyzed using SPSS 21.0. Mean and standard deviations were calculated for continuous variables. Percentages and frequencies for categorical variables were determined. Chi-square test was applied to find association between categorical variables.

## RESULTS

A total of 69 cases were retrieved from the database with a mean age of 46.97 ± 13.86 years. The demographic and clinical features of the patients are shown in Table-1. More than one-half of the patients had BMI >25kg/m2 i.e., 40 (58%). Almost 26 percent of the women had hypertension and almost one-half of the women had previous gynecological surgery. About 30 (43.5%) women had stage two, 30 (43.5%) had stage three, and the remainder had stage four. Most of the participants had concomitant surgery of the pelvis which included anti incontinence surgery (Mid-urethral slings or Burch) in 13 (18.8%), Anterior and posterior colporrhaphy in 28 (40.5%), bilateral oophorectomy in 4 (5.7%) and 1 woman (1.44%) had anal sphincteroplasty. In 33(47.8%) patients, Sacrocolpopexy and in 36 (52.2%) patients Sacrohysteropexy was performed.

**Table-I:** Demographics and Clinical characteristics.

Parameters	N (%)
Age (years)	46.97 ± 13.86
***Age group (years)***	
Less than or equal to 50 years	41 (59.4%)
More than 50 years	28 (40.6%)
Body Mass Index	
Less than or equal to 25	29 (42%)
More than 25	40 (58%)
***Menopause status***	
Yes	34 (49.3%)
No	35 (50.7%)
***Comorbidities***	
None	45 (65.2%)
Hypertension	18 (26.1%)
Diabetes Mellitus	6 (8.7%)
***Previous gynecological surgery***	
Yes	37 (53.6%)
No	32 (46.4%)
***Pre-surgery POP Q***	
Stage 2	30 (43.5%)
Stage 3	30 (43.5%)
Stage 4	9 (13%)
***Concomitant pelvic surgery***	
Yes	46 (66.6%)
No	23 (33.3%)
***Type of apical prolapse surgery***	
Sacrocolpopexy	33 (47.8%)
Sacrohysteropexy	36 (52.2%)
Total follow up (months)	22.27 ± 19.15

Intraoperative complications and clinical parameters are illustrated in Table-II. The mean length of hospitalization was 3.91 ± 0.74 days. The mean blood loss during operation was 286.76 ± 165.41 ml. The rate of severe adhesions was 8.7%.

**Table-II:** Intraoperative findings.

Parameter	N (%)
***Severe adhesions encountered***	
Yes	6 (8.7%)
No	63 (91.3%)
Estimated blood loss (ml)	286.76 ± 165.41
***Bowel rectal injury***	
Yes	1 (1.4%)
No	68 (98.6%)
Operative time (mins)	143.58 ± 54.23
***Blood transfusion***	
Yes	7 (10.1%)
No	62 (89.9%)
Hospital stay (days)	3.91 ± 0.74

Immediate complications postoperatively (within two weeks of surgery) are mentioned in Table-2. It was found that 14 (20.3%) patients had wound infection while six (8.7%) patients developed urinary tract infection.

Overall, in complications mesh erosion into vagina were found in 3/69 (4.3%) during the median 12-month follow-up, 1/36 after Sacro-hysteropexy, 2/33 after Sacro-colpopexy. One patient had a mesh-erosion into vagina within six months after Sacro-colpopexy, the main complaint was abnormal vaginal discharge. Exposed part of mesh of about 1.5cm found during speculum examination at level of vaginal apex. The exposed part of the mesh was removed trans-vaginally.

**Fig.1:**
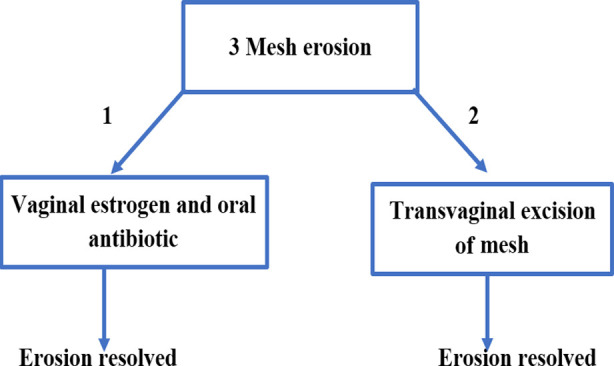
Outcomes of the management of vaginal mesh erosion.

In the next six months to twelve months postoperatively, two more patients presented with mesh erosion into vagina with frequent complaint of vaginal discharge and discomfort. One patient had bleeding at 11 months postoperatively, at which point the exposed mesh of about one cm was excised trans-vaginally, and the other patient was successfully managed on conservative treatment with topical estrogen and oral antibiotic (Fig.1). Objective evidence of POP was documented in four (5.8%), de-novo LUTS in 15 (21.7%), and sexual discomfort in five (7.2%) patients.

Illustrated mean in Table-IV is preoperative and postoperative POPDI-six scores among patients. Extremely significant improvements were observed in POPDI-six scores six months postoperatively (p=0.0001).

**Table-III:** Postoperative short-term and long-term complications.

Short term complications (two -weeks)	N (%)
Wound infection	14 (20.3%)
Urinary tract infection	6 (8.7%)
Urinary retention	1 (1.4%)
Intestinal obstruction	3 (4.3%)
Late complications (1-6 months)	
Mesh related complication	1 (1.45%)
Complaints of recurrence of Pelvic organ prolapse	3 (4.3%)
Objective evidence of Pelvic organ prolapse	1 (1.45%)
Sexual discomfort	2 (2.9%)
Need for second procedure	0
Bleeding from rectum	0
De Novo LUTS	2 (2.9%)
Late complications (6-12 months)	
Mesh related complication	2 (2.9%)
Complaints of recurrence of Pelvic organ prolapse	3 (4.3%)
Objective evidence of Pelvic organ prolapse	4 (5.8%)
Sexual discomfort	5 (7.2%)
Need for second procedure	1 (1.4%)
Bleeding per rectum	0
De Novo Lower urinary tract symptoms	15 (21.7%)

**Table-IV:** POPDI-six scores at baseline, six months, and twelve months postoperative.

Mean POPDI-6 score	Mean ± SD	Change in POPDI-6	95% C.I	p-value
Pre-operative	20.83 ± 14.02	-		
Six months post-operative	3.26 ± 7.73	17.57 ± 14.2	20.98,14.16	<0.0001
Twelve months post-operative	2.54 ± 7.7	0.73 ± 4.3	1.75,0.31	0.165

## DISCUSSION

We report a low incidence of mesh related complications of 4.3%, at median follow up of 12 months from a cohort of 69 patients who underwent sacrohysteropexy and sacrocolpopexy, notably there were no case of mesh erosion into abdominal viscera. Due to the heterogeneity in reporting mesh issues, it is challenging to compare our data to those in the literature for abdominal mesh. In contrast to a study by Tijdink MM et al., which reported mesh-related complications in 27% of women with POP repair, we reported a much lower proportion of 4.3%.^14^ This could be due to several reasons. For instance, a surgeon’s vast experience and optimum postoperative management can be attributed to a lower rate in our study.

The advantages of transabdominal techniques for apical prolapse repair include more accurate anchoring of the vaginal apex and minimal change in vaginal anatomy. Overall, in our study few patients reported sexual discomfort and in most of them, de novo dyspareunia after abdominal surgery had a low rate of 7.2%. Our finding is similar to a study in the UK that reported Dyspareunia in 6% on follow-up.^15^ Likewise, Dandolu et al. reported Dyspareunia at 2.2%.^16^ Reoperation rates for prolapse in any compartment in our study is 1.4%. This is lower than findings by Pedersen KD et al., in which 35% of women had a repeat procedure.^17^ This difference can be explained by patients being followed for a longer time of seven years in the study done by Pedersen KD et al., compared to our study where the follow-up period is one year.^17^ Additionally, different hospitals have distinct management plans that may contribute to diverse results.

Urinary tract infections were low in our study unlike a study by Fatton et al., which reported a higher percentage of 11.8% of patients developing UTI.^18^ UTI rate in our study was comparable to findings by Kaufman et al., who reported a percentage of 8.6%.^19^ On the other hand, our findings are different from a retrospective study by Wang et al. which reported UTIs in 6% of women.^20^ During 12 month follow up, De novo lower urinary tract symptoms which has been reported to occur postoperatively in our study was 21.7%. The common symptoms were increase urinary frequency and urgency; we didn’t encounter any patient who developed de novo stress urinary incontinence. This finding is different from a study in which 15.3% of all participants who underwent ASC without incontinence surgery developed de novo SUI.^21^

Around 20.3 % of patients developed wound infection in the first two weeks after surgery in our study. This is different from a study done in Denmark, which reported wound infection in 0.5% of patients after surgery.^22^ Likewise, a case series study carried out in the UK reported a lower wound infection rate of 2%.^23^ This is an alarming situation that requires further investigation. Within the first 12 months of follow-up, none of our patients experienced gastrointestinal issues that required a readmission, surgery, or hospitalization. These findings were consistent with those published in the literature.^24^

According to our study, 1.2% of women experienced urinary retention in the first two weeks after surgery. In contrast to this, Fatton et al. reported higher urinary retention of 11.8%.^18^ Adhesions were encountered in 8.7% of women in our study. This is different from a study conducted in France in which an adhesion rate of 4.5% was observed.^25^

In our study, 4.3% of women complained of subjective recurrence of prolapse. This finding is in line with a study done in China where the subjective recurrence was 5%.^26^ Subsequently, a study by Unger et al. reports a higher recurrence rate of 43%.^27^ A reason for this higher recurrence in studies done by Unger et al and Pedersen KD et al. is a longer follow-up period of six and seven years respectively, while in our study patients were followed for one year.^17^,^27^

### Limitations

Despite the apparent strengths of the study, we did counter some limitations. For instance, the data presented in the current study is from a single center. Efficacy and safety profiles, as well as patient outcomes, may vary from one institute to the other or from one surgeon to the other. This may limit the generalizability of the findings.

## CONCLUSION

Our findings present that Sacro-colpopexy and Sacro-hysteropexy both substantially improved symptoms of POP in patients and the complication rate was quite low. The present study signifies the use of abdominally placed mesh in patients with pelvic organ prolapse indicating significant improvement in POP-associated symptoms postoperatively. This lends credence to the fact that non-absorbable abdominal mesh for apical prolapse surgery bears good clinical outcome. However, we did report a significant proportion of patients with post-operative wound infection which needs further exploration.

### Author’s contributions:

This work was carried out in collaboration among all authors.

**SA** designed the study, wrote the protocol, data collection, and wrote manuscript,

**UK** managed the analysis and contribution in manuscript writing.

**NC** and **SM** managed the literature searches and review of manuscript.

**UK and SA** are responsible and accountable for the accuracy and integrity of the work.

All author’s read and approved the final manuscript.
